# Effect of pulmonary vein isolation on atrial fibrillation recurrence after accessory pathway ablation in patients with Wolff‐Parkinson‐White syndrome

**DOI:** 10.1002/clc.23470

**Published:** 2020-10-01

**Authors:** Jin‐Tao Wu, Dan‐Qing Zhao, Fei‐Fei Li, Lei‐Ming Zhang, Juan Hu, Xian‐Wei Fan, Guang‐Ling Hu, Hai‐Tao Yang, Li‐Jie Yan, Jing‐Jing Liu, Xian‐Jing Xu, Shan‐Ling Wang, Ying‐Jie Chu

**Affiliations:** ^1^ Heart Centre of Henan Provincial People's Hospital, Central China Fuwai Hospital, Central China Fuwai Hospital of Zhengzhou University Zhengzhou China; ^2^ Department of Cardiology Henan University People's Hospital, Henan Provincial People's Hospital Zhengzhou China; ^3^ Human Resource Department The Third Affiliated Hospital of Zhengzhou University Zhengzhou China

**Keywords:** accessory pathway ablation, advanced interatrial block, atrial fibrillation, pulmonary vein isolation, Wolff‐Parkinson‐White syndrome

## Abstract

**Background:**

Although successful ablation of the accessory pathway (AP) eliminates atrial fibrillation (AF) in some of patients with Wolff‐Parkinson‐White (WPW) syndrome and paroxysmal AF, in other patients it can recur.

**Hypothesis:**

Whether adding pulmonary vein isolation (PVI) after successful AP ablation effectively prevents AF recurrence in patients with WPW syndrome is unknown.

**Methods:**

We retrospectively studied 160 patients (102 men, 58 women; mean age, 46 ± 14 years) with WPW syndrome and paroxysmal AF who underwent AP ablation, namely 103 (64.4%) undergoing only AP ablation (AP group) and 57 (35.6%) undergoing AP ablation plus PVI (AP + PVI group). Advanced interatrial block (IAB) was defined as a P‐wave duration of >120 ms and biphasic (±) morphology in the inferior leads, using 12‐lead electrocardiography (ECG).

**Results:**

During the mean follow‐up period of 30.9 ± 9.2 months (range, 3‐36 months), 22 patients (13.8%) developed AF recurrence. The recurrence rate did not differ in patients in the AP + PVI group and AP group (15.5% vs 10.5%, respectively; *P* = .373). Univariable and multivariable Cox regression analyses showed that PVI was not associated with the risk of AF recurrence (hazard ratio, 0.66; 95% confidence interval, 0.26‐1.68; *P* = .380). In WPW patients with advanced IAB, the recurrence rate was lower in patients in the AP + PVI group vs the AP group (90% vs 33.3%, respectively; *P* = .032).

**Conclusions:**

PVI after successful AP ablation significantly reduced the AF recurrence rate in WPW patients with advanced IAB. Screening of a resting 12‐lead ECG immediately after AP ablation helps identify patients in whom PVI is beneficial.

## 1 INTRODUCTION

Atrial fibrillation (AF) occurs frequently in patients with Wolff‐Parkinson‐White (WPW) syndrome, with a reported incidence of 9% to 38%.^1^, ^2^, ^3^, ^4^ Although previous studies have reported a decreased incidence of AF after successful elimination of the accessory pathway (AP),^5^, ^6^ paroxysmal AF frequently recurs in some patients with WPW syndrome despite successful AP elimination.^3^, ^4^, ^7^, ^8^, ^9^ Our recent study found that the presence of advanced interatrial block (IAB) and age >50 years were associated with a higher risk of AF recurrence after AP ablation in WPW patients.^10^ For patients at high risk of AF recurrence, additional preventive interventions are required. Currently, pulmonary vein intervention (PVI) is a widely accepted treatment for symptomatic paroxysmal AF.^11^ However, it is unknown whether additional PVI after successful AP ablation effectively prevents AF recurrence in patients with WPW syndrome, especially in patients at high risk of AF recurrence. Thus, the purpose of this retrospective study was to investigate the efficacy of additional PVI after AP ablation in preventing AF recurrence in all patients with WPW syndrome and paroxysmal AF, and in patients at high risk of AF recurrence, identified by advanced IAB or age >50 years.

## 2 METHODS

### 2.1 Patients

Consecutive patients with overt or intermittent WPW syndrome who were hospitalized at Henan Provincial People's Hospital and Fuwai Central China Cardiovascular Hospital for radiofrequency ablation between January 2013 and September 2018 were retrospectively reviewed. The inclusion criteria were (a) at least one documented episode of AF before ablation; (b) undergoing AP ablation, with or without catheter ablation for AF during the same session; (c) successful AP ablation, defined as the elimination of AP conduction by demonstrated atrial and ventricular pacing even after isoproterenol infusion; and (d) available records of a post‐ablation, 12‐lead electrocardiogram (ECG). The exclusion criteria were (a) repeated ablations; (b) previous cardiac surgery, congenital heart disease, or serious valvular heart disease; and (iii) thyroid dysfunction on admission (abnormal free thyroxine or thyroid‐stimulating hormone level). We divided patients into two groups: the “AP group” constituted patients who underwent AP ablation without PVI, and the “AP + PVI group” constituted patients who underwent AP ablation and PVI during the same session. The study protocol conformed to the ethical guidelines of the Declaration of Helsinki. All patients were informed about the investigational nature of the catheter ablation procedure and provided written informed consent to undergo the procedure. The study protocol was approved by the local institutional review board, and the requirement for informed consent for this study was waived because of its retrospective nature.

### 2.2 Electrophysiological study and catheter ablation

#### 2.2.1 AP ablation

All patients in both the AP group and AP + PVI group underwent successful AP ablation. A two‐dimensional mapping system or the CARTO three‐dimensional electroanatomical mapping system (Biosense Webster, Diamond Bar, California) was used in the ablation procedures. Patients underwent an electrophysiological study after all antiarrhythmic drugs had been discontinued for at least five half‐lives and before AP ablation was performed. Three 6‐French multipolar electrode catheters (Biosense Webster) were introduced percutaneously into the bilateral femoral veins for electrophysiological studies. The catheters were positioned in the high right atrium, across the tricuspid valve, to record the His‐bundle ECG, and in the right ventricular apex. Another 6‐French multipolar electrode catheter (Biosense Webster) was advanced from the right internal jugular vein and positioned in the coronary sinus. A retrograde aortic approach or the trans‐septal approach was used for left‐sided pathways. Right‐sided pathways were approached via the femoral veins. The locations of the APs were determined from the position of the catheter at a successfully ablated site in the left oblique fluoroscopic view.^12^

#### 2.2.2 AF ablation

Patients in the “AP + PVI group” underwent additional PVI immediately after AP ablation. Additional PVI was offered to patients, with their consent and at the operator's discretion. The PVI procedure was performed in the post‐absorptive state under conscious sedation. We used the technique of circumferential PV ablation guided by three‐dimensional left atrial (LA) mapping, which was described previously in detail.^13^ Briefly, the LA was explored using the trans‐septal approach. The LA geometry was reconstructed with a 3.5‐mm tip Thermocool SmartTouch catheter (Biosense Webster) in the CARTO three‐dimensional electroanatomical mapping system. Continuous irrigated radiofrequency ablation was performed along each PV antrum to encircle the ipsilateral PVs (target temperature, 43°C; maximum power, 35 W; infusion rate, 17 mL/min). The procedural endpoints were completeness of continuous circular lesions and electrical isolation of all PVs as identified by a decapolar circumferential mapping catheter (Lasso; Biosense Webster).

### 2.3 ECG analysis

**FIGURE 1:**
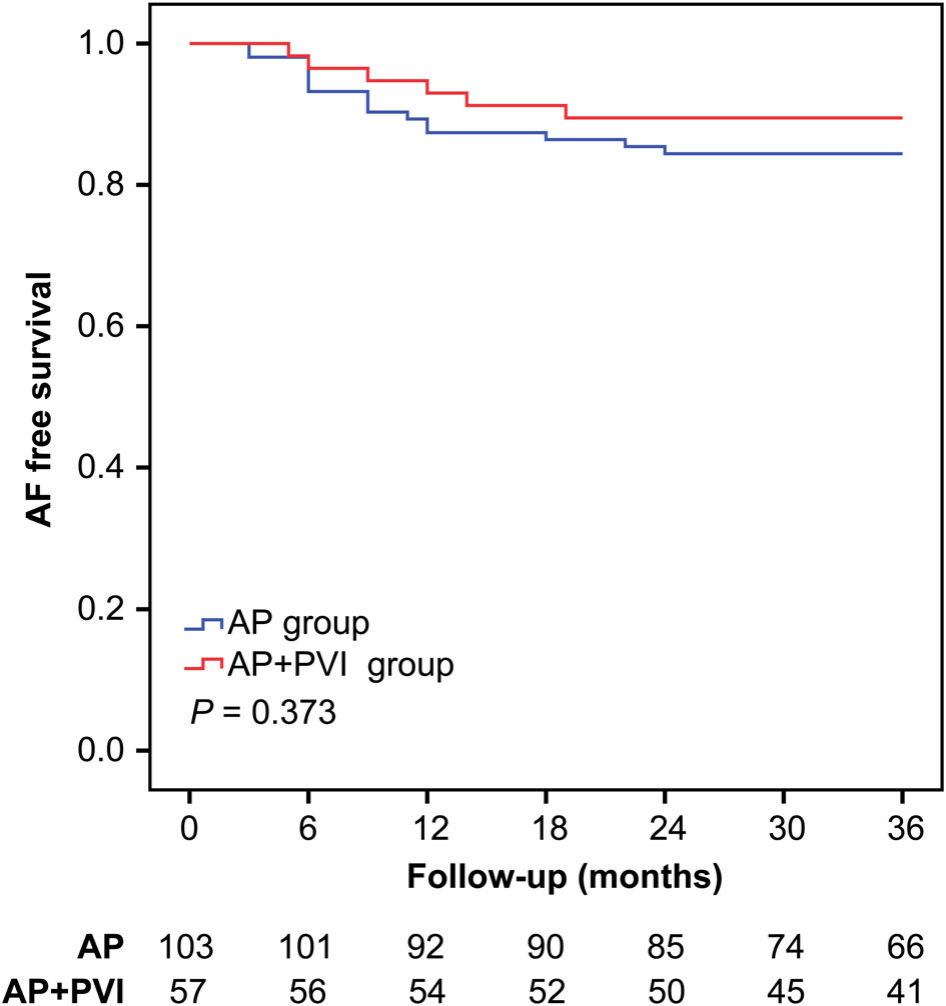
Kaplan‐Meier curves showing atrial fibrillation (AF) recurrence stratified by whether patients underwent simultaneous additional pulmonary vein isolation (PVI). The AF recurrence rate was not significantly different between patients who underwent accessory pathway (AP) ablation plus PVI and patients who underwent AP ablation only (15.5% vs 10.5%, respectively; *P* = .373)

In all patients, a resting 12‐lead ECG in sinus rhythm (high‐pass filter, 0.05 Hz; low‐pass filter, 150 Hz; 25 mm/s; 10 mm/mv) was obtained immediately after the AP ablation procedure. All ECGs were transmitted electronically using Vhcloud Network Solution (Vales and Hills Biomedical Tech. Ltd., Beijing, China) for storage at the ECG Core Laboratory of Henan Provincial People's Hospital and Fuwai Central China Cardiovascular Hospital. ECGs were manually analyzed on a computer screen using digital calipers with scanning at 300 dots per square inch and fourfold image amplification. P‐waves were measured manually using digital calipers for all 12 ECG leads to identify the longest P‐wave duration, as previously described.^14^ Advanced IAB was defined as a P‐wave of >120 ms accompanied by a biphasic (±) morphology in the inferior leads (Figure 1).^15^ The ECG analysis was performed independently by two observers who were blinded to the patient details, and any differences between the observers were resolved by consensus.

### 2.4 Patient follow‐up

After the ablation procedure, all patients were required to visit their physician at 3, 6, and 12 months, and every year, thereafter. A 12‐lead ECG and 24‐hours Holter recording were obtained at every visit. If a patient exhibited any symptoms suggesting tachyarrhythmia, namely palpitations, syncope, or dizziness, a new ECG and 24‐hours Holter recording were obtained. For any event reported between visits, the patient's medical records were retrieved and reviewed. All patients included in the study were followed up until AF occurrence or 3 years after the procedure or June 2020, whichever came first. AF recurrence was defined as confirmed AF lasting more than 30 seconds, as documented by ECG or Holter recordings.^16^ We implemented a 3‐month blanking period for outcomes assessed across both groups, in this study.

### 2.5 Statistical analysis

All analyses were performed using statistical software (SPSS version 24.0; IBM Corp., Armonk, New York). Continuous data are presented as mean ± SD and were compared using an unpaired independent‐samples *t*‐test or one‐way analysis of variance. Categorical variables are presented as a percentage of the group total and were compared using the *χ* ^2^ test or Fisher's exact test, as appropriate. A Kaplan‐Meier estimation with a log‐rank test was performed for unadjusted analysis of the impact of the additional PVI procedure on AF recurrence. Cox proportional hazards regression was used to examine the risk of recurrence. All probability values were two‐sided, and values of *P* < .05 were considered statistically significant.

## 3 RESULTS

We enrolled 160 patients in this study, namely 103 patients in the AP group and 57 patients in the AP + PVI group. Advanced IAB was detected in 16 (10.0%) patients, namely 10 patients in the AP group and 6 patients in the AP + PVI group. Patients' baseline characteristics in both groups are shown in Table 1.

**TABLE 1:** Baseline characteristics of the study population

	AP group (n = 103)	AP + PVI group (n = 57)	*P* value
Age, years	44 ± 16	48 ± 10	.112
Age > 50	41(39.8%)	23(40.4%)	.946
Male, n (%)	70 (68.0%)	32 (56.1%)	.136
AF duration, months	9.0 ± 7.6	9.9 ± 6.4	.484
DM, n (%)	13(12.6%)	7(12.3%)	.950
Hypertension, n (%)	20(19.4%)	14(24.6%)	.446
CAD, n (%)	7 (6.8%)	4 (7.0%)	1.00
CHA_2_DS_2_‐VASc score	0.8 ± 1.1	0.7 ± 0.9	.624
Left atrial diameter, mm	36.9 ± 4.2	37.9 ± 4.1	.173
LVEF, %	65.1 ± 5.4	64.7 ± 5.6	.674
aIAB, n(%)	10(9.7%)	6(10.5%)	.869
Intermittent WPW syndrome, n (%)	10(9.7%)	4(7.0%)	.776
Presence of retrograde conduction via AP, n (%)	100 (97.1%)	54(94.7%)	.667
Single left‐sided AP, n (%)	60 (58.3%)	35(61.4%)	.698
Single right‐sided AP, n (%)	38 (36.9%)	19(33.3%)	.652
Multiple APs, n (%)	5 (4.9%)	3(5.3%)	1.00
Follow‐up, months	30.3 ± 9.7	32.0 ± 8.2	.262

Abbreviations: aIAB, advanced interatrial block; AF, atrial fibrillation; AP, accessory pathway; CAD, coronary artery disease; CHA_2_DS_2_‐VASc, coronary artery disease, congestive heart failure, hypertension, age ≥75 years, diabetes mellitus, stroke/transient ischemic attack, vascular disease, age 65 to 74 years, sex category; DM, diabetes mellitus; LVEF, left ventricular ejection fraction; PVI, pulmonary vein isolation; WPW, Wolff‐Parkinson‐White.

During the mean follow‐up period of 30.9 ± 9.2 months (range, 3‐36 months), 22 patients (13.8%) developed AF recurrence. The recurrence rate was 15.5% and 10.5% in the AP group and AP + PVI group, respectively. Kaplan‐Meier analysis showed that freedom from recurrent AF was not significantly different between the AP group and AP + PVI group (log‐rank test, *P* = .373; Figure 1).

Univariable Cox proportional hazards regression analysis showed that, for the overall study population after the ablation procedure, age >50 years; diabetes mellitus; coronary artery disease; CHA_2_DS_2_‐VASc (congestive heart failure, hypertension, age ≥75 years, diabetes mellitus, stroke/transient ischemic attack, vascular disease, age 65‐74 years, sex category) score; LA diameter; and advanced IAB were associated with the risk of recurrence. Multivariable analysis identified advanced IAB (hazard ratio, 5.50; 95% confidence interval [CI], 2.29‐13.22; *P* < .001), and age >50 years (hazard ratio, 10.02; 95% CI, 2.20‐45.59; *P* = .003) as independent predictors of AF recurrence (Table 2). The PVI procedure was not associated with the risk of AF recurrence in univariable and multivariable Cox regression analyses.

**TABLE 2:** Predictors of atrial fibrillation recurrence after the ablation procedure in the total study population

	Univariate Cox regression			Multivariate Cox regression		
	HR	95% CI	*P* value	HR	95% CI	*P* value
Age>50	17.11	4.00‐73.23	<.001	10.02	2.20‐45.59	.003
Male, n (%)	1.23	0.50‐3.00	.658			
AF duration, months	1.01	0.96‐1.07	.682			
DM, n (%)	6.20	2.65‐14.54	<.001			
Hypertension, n (%)	2.14	0.90‐5.11	.085			
CAD, n (%)	4.82	1.78‐13.07	.002			
CHA_2_DS_2_‐VASc score	2.07	1.55‐2.77	<.001			
Left atrial diameter, mm	1.20	1.08‐1.35	.001			
aIAB, n(%)	12.75	5.49‐29.62	<.001	5.50	2.29‐13.22	<.001
Single left‐sided AP, n (%)	1.21	0.51‐2.88	.670			
Multiple APs, n (%)	0.90	0.12‐6.66	.915			
PVI procedure	0.66	0.26‐1.68	.380			

Abbreviations: aIAB, advanced interatrial block; AF, atrial fibrillation; AP, accessory pathway; CAD, coronary artery disease; CHA_2_DS_2_‐VASc, congestive heart failure, hypertension, age ≥75 years, diabetes mellitus, stroke/transient ischemic attack, vascular disease, age 65 to 74 years, sex category; CI, confidence interval; DM, diabetes mellitus; HR, hazard ratio; PVI, pulmonary vein isolation.

**FIGURE 2:**
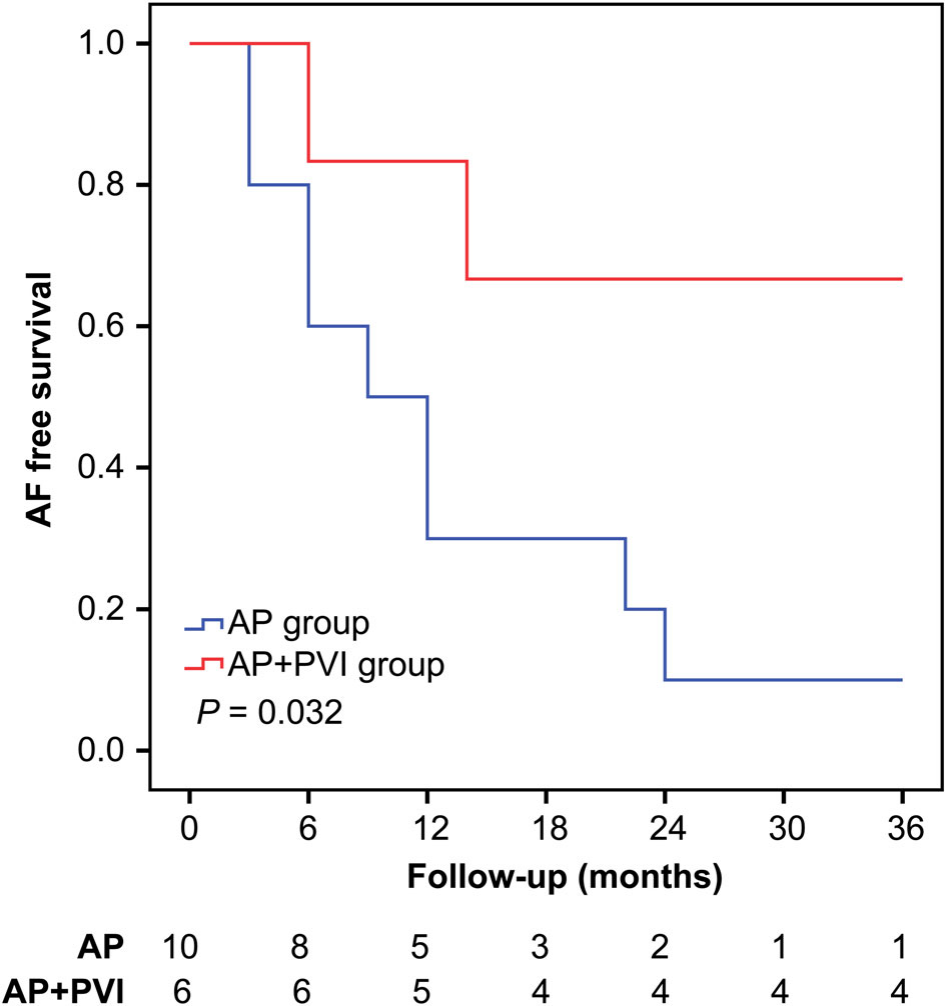
Kaplan‐Meier curves showing atrial fibrillation (AF) recurrence stratified by whether the subgroup of patients with advanced interatrial block (IAB) underwent additional simultaneous pulmonary vein isolation (PVI). The AF recurrence rate was lower in patients who underwent accessory pathway (AP) ablation plus PVI than those who underwent AP ablation only (90% vs 33.3%, respectively; *P* = .032)

**FIGURE 3:**
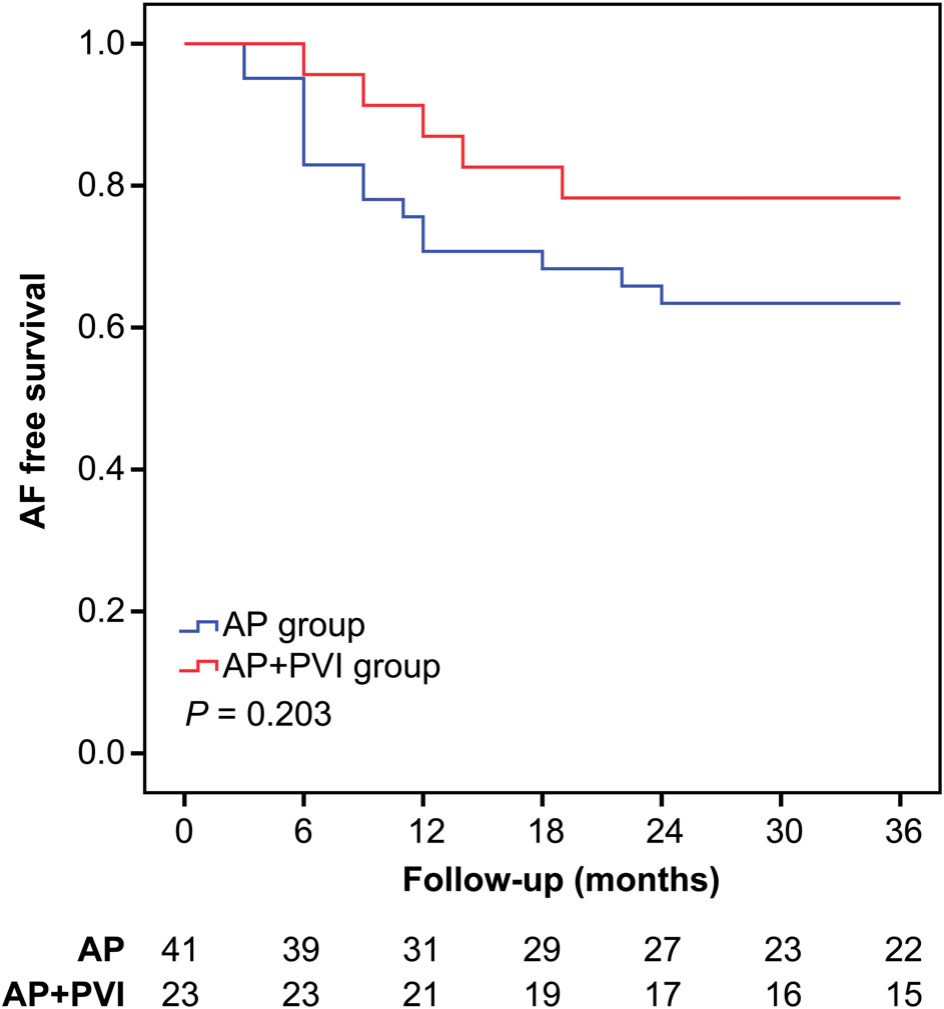
Kaplan‐Meier curves showing atrial fibrillation (AF) recurrence stratified by whether the subgroup of patients aged >50 years underwent additional simultaneous pulmonary vein isolation (PVI). The AF recurrence rate was not significantly different between patients who underwent accessory pathway (AP) ablation plus PVI and patients who underwent AP ablation only (36.6% vs 21.7%, respectively; *P* = .203)

In this study, we assessed the effect of PVI on AF recurrence in patients at high risk for AF recurrence. Among patients with advanced IAB, Kaplan‐Meier analysis showed that the recurrence rate was lower in patients in the AP + PVI group than in the AP group (90% vs 33.3%, respectively; *P* = .032) (Figure 2). Among patients aged >50 years, Kaplan‐Meier analysis showed that the rate of AF recurrence was not significantly different between the AP group and the AP + PVI group (36.6% vs 21.7%, respectively; *P* = .203; Figure 3).

## 4 DISCUSSION

The main findings in this study were as follows: (a) PVI after successful AP ablation significantly reduced AF recurrence in the subgroup of WPW patients with advanced IAB and (b) PVI did not reduce AF recurrence in nonselective WPW patients and in patients aged >50 years.

Previous studies have shown that patients with WPW syndrome have a high incidence of paroxysmal AF.^1^, ^2^, ^3^, ^4^ The potential mechanisms responsible for the occurrence of AF in patients with WPW syndrome is thought to be non‐PV related, and are attributed to spontaneous degeneration of atrioventricular reciprocating tachycardia into AF, electrical properties of the AP, effects of AP on atrial architecture, and intrinsic atrial muscle vulnerability.^17^, ^18^ However, Derejko et al^8^ recently suggested a potential role of the PVs also in patients with WPW syndrome and AF. Because successful ablation of the AP eliminates AF in some patients but not in other patients,^3^, ^4^, ^7^, ^8^, ^9^ it is suggested that AP‐dependent and AP‐independent paroxysmal AF exist in patients with WPW. PVI is a widely accepted treatment option for symptomatic paroxysmal AF.^11^ Although the PV is reportedly involved in the development of paroxysmal AF in patients with WPW syndrome,^8^ atrial substrate abnormalities are thought to be mainly associated with the risk of AF recurrence.^4^, ^9^ Thus, it is unknown whether additional PVI immediately after AP ablation effectively prevents AF recurrence in these patients. A recent study showed that adding PVI after successful ablation of paroxysmal supraventricular tachycardia (29 of the patients had APs) reduced the AF recurrence rate, but the difference did not reach statistical significance.^19^ Kawabata et al^6^ compared the efficacy of AP ablation alone and additional AF ablation on accompanying AF and found that adding PVI did not improve the freedom from residual AF compared with AP ablation alone in all patients with WPW syndrome and AF; however, adding PVI significantly reduced AF recurrence in a subgroup of patients with brain natriuretic peptide (BNP) concentrations >40 pg/mL. The authors concluded that screening BNP concentrations would identify the subset of patients in whom additional PVI would be beneficial. However, BNP concentrations might fluctuate because BNP is affected by many factors.

In the present study, we evaluated the efficacy of additional PVI after AP ablation in preventing AF recurrence, and in a subgroup of patients with advanced IAB or aged >50 years. Our study showed that additional PVI after AP ablation did not reduce the AF recurrence rate in nonselective WPW patients, which is in accordance with the findings of Kawabata et al's study. In addition, we found that PVI significantly reduced the AF recurrence rate in the subgroup of WPW patients with advanced IAB. Our findings suggest that, for patients at high risk of AF recurrence identified by advanced IAB, additional PVI after AP ablation can be considered effective to prevent AF recurrence. Screening of a resting 12‐lead ECG immediately after AP ablation helps identify patients in whom PVI should be performed. Furthermore, our results also strengthen the consideration that PVI may not be necessary in nonselective patients with WPW syndrome and AF.

Because several studies showed that age >50 years was an independent predictor of AF recurrence after AP ablation,^3^, ^4^, ^7^, ^10^ we examined the efficacy of additional PVI after AP ablation in preventing AF recurrence in these subgroup of patients, in the present study. We found that additional PVI did not significantly reduce AF recurrence in these patients. This information suggests that, although it was an independent predictor of AF recurrence after successful AP, age >50 years may not identify the subset of WPW patients in whom PVI in addition to AP ablation would be beneficial.

## 5 LIMITATIONS

Several limitations of our study should be considered. First, the small sample size may have introduced statistical bias. Further studies of larger numbers of patients are needed to further evaluate the effects of PVI on AF recurrence after AP ablation in patients with WPW syndrome. Second, it is not possible to identify advanced IAB before AP ablation in patients with WPW syndrome because the delta wave overshadows the point at which the P‐wave ends. Additionally, P‐wave duration was measured based on the ECG obtained immediately after the ablation procedure; thus, residual atrial injury from the ablation procedure may have affected the P‐wave duration. Finally, the generalizability of our findings may be limited by the single‐center, retrospective, observational approach.

## 6 CONCLUSION

Additional PVI after successful AP ablation may not reduce AF recurrence in nonselective WPW patients; however, the AF recurrence rate was reduced significantly in the subgroup of WPW patients with advanced IAB. Screening of a resting 12‐lead ECG immediately after AP ablation helps identify patients in whom additional PVI following AP ablation would be beneficial.

## CONFLICT OF INTEREST

The authors declare that there is no conflict of interest.

## Data Availability

Data available on request from the authors.
